# Abnormal expression of the Notch and Wnt/β-catenin signaling pathways in stem-like ALDH^hi^CD44^+^ cells correlates highly with Ki-67 expression in breast cancer

**DOI:** 10.3892/ol.2015.2942

**Published:** 2015-02-09

**Authors:** JUNWEI CUI, PENG LI, XIAOLING LIU, HUI HU, WEI WEI

**Affiliations:** Department of Breast Surgery, Peking University Shenzhen Hospital, Shenzhen, Guangdong 518036, P.R. China

**Keywords:** breast cancer, stem-like cell, signaling pathway, immunohistochemical

## Abstract

Previous studies have reported that breast cancer stem cells may be closely associated with tumor metastasis, recurrence, and even the failure of chemotherapy and radiotherapy. The aim of the present study was to investigate whether important cell signaling pathways associated with drug resistance are activated in stem-like acetaldehyde dehydrogenase (ALDH)^hi^ cluster of differentiation (CD)44^+^ cells, and to analyze the association between ALDH^hi^CD44^+^ cells and specific pathological features. ALDH^hi^CD44^+^ cells and non-stem-like ALDH^low^CD44^+^ cells were separated from MDA-MB-231 cells by fluorescence-activated cell sorting, and the mRNA expression levels of Notch1 and β-catenin were estimated by performing quantitative polymerase chain reaction in the stem-like and non-stem-like cells. Line correlation analysis was used to evaluate the correlation between an immunohistochemical panel of estrogen receptor (ER), progesterone receptor (PR), human epidermal growth factor receptor 2 (HER2) and Ki-67, and ALDH^hi^CD44^+^ cells from patients with invasive breast carcinoma. The mRNA levels of Notch1 and β-catenin were significantly higher in the ALDH^hi^CD44^+^ cells compared with those in the ALDH^low^CD44^+^ cells (P<0.05); furthermore, the present study determined a high correlation (P<0.05) between the ALDH^hi^CD44^+^ cells and Ki-67 expression (P=0.007), but no correlation (P≥0.05) with ER (P=0.065), PR (P=0.107) and HER2 (P=0.050). Overall, these data clearly indicate that ALDH^hi^CD44^+^ cells may serve as novel diagnostic and prognostic factors in breast cancer.

## Introduction

Breast cancer stem cells, which are capable of inducing tumor recurrence following chemotherapy or radiotherapy treatment, are regarded as a future novel therapeutic target of breast cancer (1–3). Recent studies demonstrated that reducing the properties of these stem cells resulted in observable suppression of their tumor sphere-forming capacity *in vitro* and *in vivo* (4). Furthermore, according to the cancer stem cell model, these stem cells or progenitor cells deregulate the typically tightly regulated process of self-renewal, causing resistance to chemotherapy and radiation treatment strategies in breast cancer (5). Acetaldehyde dehydrogenase 1 (ALDH1) is one of the important markers of breast cancer stem cells. ALDH1 is a member of a group of isoenzymes, and ALDH^hi^ cells exhibit a greater ability to form tumors compared with ALDH^low^ cells (6). Furthermore, cluster of differentiation (CD)44^+^, CD133^+^ and CD24^−/low^ are regarded as important surface markers of breast cancer stem cells. Croker and Allan (7) identified that relative to ALDH^low^CD44^−^ cells, ALDH^hi^CD44^+^ cells in mice and in *in vitro* experiments demonstrated stronger invasion and metastatic ability, and inhibition of ALDH activity could reduce the chemotherapy and radiotherapy resistance of the ALDH^hi^CD44^+^ cells.

Notch and Wnt/β-catenin signaling pathways are involved in the regulation of cellular gene expression and metabolism (8,9). Notch signaling, which is frequently dysregulated, and particularly overactivated, induces the development of a number of different cancer types and confers a survival advantage on tumors, resulting in poorer outcomes for patients (10). Furthermore, it has been determined that Notch1 of the Notch signaling pathway may affect chemotherapeutic agent resistance in head and neck squamous cell carcinoma, ovarian cancer and gastric cancer (11). In addition, a previous study identified that drug resistance gene expression levels increased in the colon cancer as β-catenin expression levels increased (12). Alternative studies have demonstrated that β-catenin may increase the resistance of tumor cells by activating matrix metalloproteinase 9 and 12 (13). Thus, inhibiting the Notch and Wnt/β-catenin signaling pathways may be successful in controlling cancer cell growth in conjunction with the application of standard chemotherapy.

According to the immunohistochemical (IHC) staining, the different subtypes of breast cancer exhibit different efficacies in response to treatment with chemotherapy, radiotherapy and endocrinotherapy. The selection of therapy for breast cancer relies on human epidermal growth factor receptor-2 (HER2), estrogen receptor α (ER), progesterone receptor (PR) and Ki-67 protein expression levels. For example, the treatment of HER2^+^ breast cancer with trastuzumab has markedly improved the outcome of this disease, but not the outcome of HER2^−^ breast cancer (14). Furthermore, patients exhibiting ER and PR negativity have a poor outcome, as they are insensitive to endocrinotherapy, for example tamoxifen (15). Assessment of the Ki-67 proportion of cells has become the most widely used method for predicting tumor cell proliferation rates and resistance to chemotherapy or endocrine therapy in tumor samples (16). However, additional analytical and clinical validation is required to determine whether specific correlations exist between cancer stem cells and IHC expression levels in breast cancer.

Our previous studies identified associations between breast cancer stem cells, ER-negative expression and distant metastasis (17), as well as a marked correlation between the detection of ALDH^hi^ cells in patients’ peripheral blood and high-grade invasive breast cancer (18). The aim of the present study was to determine whether two important cell signaling pathways, the Notch and Wnt/β-catenin pathways, are activated in stem-like ALDH^hi^CD44^+^ cells. To the best of our knowledge, the current study is the first study to assess the association between ALDH^hi^CD44^+^ cells and ER, PR, HER2 and Ki-67 protein expression levels using a primary culture of breast cancer cells.

## Materials and methods

### Cell culture and fluorescence-activated cell sorting

MDA-MB-231 breast cancer cells were obtained from the American Type Culture Collection (Manassas, VA, USA) and maintained in Dulbecco’s modified Eagle’s medium supplemented with 10% fetal bovine serum (FBS; Gibco Life Technologies, Carlsbad, CA, USA) at 37°C in an atmosphere of 5% CO_2_. The stem-like ALDH^hi^CD44^+^ cells and non-stem-like ALDH^low^CD44^+^ cells were separated from the MDA-MB-231 cell culture using fluorescence-activated cell sorting with an ALDEFLUOR™ assay kit (Stemcell Technologies, Inc., Vancouver, BC, Canada), and monoclonal fluorescence-conjugated rat anti-mouse antibodies against CD44-phycoerythrin (PE; 1:100) and immunoglobulin (Ig) G1-PE (1:100; Abcam, Cambridge, UK), according to the manufacturer’s instructions. Briefly, the cells were incubated in ALDEFLUOR assay buffer containing ALDH substrate. In each experiment, a sample of cells was incubated with 50 mmol/l diethylaminobenzaldehyde (a specific ALDH inhibitor) at 37°C for 45 min, for use as the negative control. Subsequently, the CD44 and IgG1 antibodies were added with ALDH into negative control Eppendorf tubes at a temperature of 4°C for 30 min. Finally, all the cells were resuspended with buffer for detection by flow cytometry.

### Extraction of total RNA and quantification of gene expression levels by quantitative polymerase chain reaction (qPCR)

Total RNA was extracted from the ALDH^hi^CD44^+^ and ALDH^low^CD44^+^ cells using an RNeasy Plus Micro kit 50 (Qiagen, Hilden, Germany). The specific primer sequences were as follows: Forward, 5′-ACCCAGAAGACTGTGGATGG-3′ and reverse, 5′-CCACCCTGTTGCTGTAGCC-3′ for GAPDH; forward, 5′-CGCCTTTGTGCTTCTGTTCTTC-3′ and reverse, 5′-CCCCACTCATTCTGGTTGTCG-3′ for Notch1; and forward, 5′-CAATCCAACAGTAGCCTTTATCAG-3′ and reverse, 5′-GCCAAGTGGGTGGTATAGAGG-3′ for β-catenin. The conditions used were 40 cycles of 30°C for 10 min, 42°C for 15 min, 9°C for 5 min and 5°C for 5 min, GAPDH was selected as the reference gene and the specificity of the PCR products was analyzed using dissociation curves. The relative RNA expression values of the transcripts were calculated using the cycle threshold (Ct) and were defined using the following equations: Relative RNA expression = 2^−ΔCt^ if ΔCt = Ct1 − Ct0, where Ct1 is the objective gene and Ct0 is GAPDH.

### Primary culture of breast cancer cells

The fresh tumor and healthy tumor-adjacent tissue samples of 24 invasive breast cancer patients, who had not undergone chemotherapy prior to surgery, were collected during surgery at Peking University Shenzhen Hospital (Shenzhen, China), and the primary culture of the breast cancer cells was obtained by employing specific enzymes, including a type IV collagen enzyme, hyaluronidase and a DNA enzyme (Sigma-Aldrich, St. Louis, MO, USA), to digest the tissues in a sterile environment at a temperature of 37°C for 4–6 h. Following digestion, the cells were collected in a strainer (mesh size, 40 μm; Corning Inc., Corning, NY, USA) and centrifuged at 250 × g for 5 min. The cells were cultured in DMEM with 10% FBS for 10–15 days prior to analysis by flow cytometry. This study was approved by the ethics committee of Peking University Shenzhen Hospital and written informed consent was obtained from all patients.

### IHC staining

IHC analysis of the tumor samples was performed by two pathologists in Peking University Shenzhen Hospital (Shenzhen, China) who had no prior knowledge of the patient outcome. ER, PR and HER2 expression levels were scored as −, +, ++ or +++, as described previously (15). HER2 status was initially determined by IHC analysis; in cases determined to be borderline (++) by immunohistochemistry, fluorescence *in situ* hybridization (FISH) was subsequently employed for the genetic testing of HER2 expression levels. Ki-67 staining was also performed in the Department of Pathology; the percentage of total cells that stained positive for Ki-67 was reported as the Ki-67 index. One case was not assessable due to an unsatisfactory staining result.

### Statistical analysis

SPSS version 13.0 (SPSS, Inc., Chicago, IL, USA) was used to analyze the differences in the mRNA expression levels of Notch1 and β-catenin in the ALDH^hi^CD44^+^ and ALDH^low^CD44^+^ cells. Comparisons between the groups were performed using the χ^2^ test for continuous variables and data were represented as the mean ± standard error of the mean. The correlation between the contents of the stem-like ALDH^hi^CD44^+^ cells, and ER, PR, HER2 and Ki-67 expression levels were analyzed using linear correlation analysis. Furthermore, a Student’s t-test was used for comparison between the two groups. P<0.05 was considered to indicate a statistically significant difference between the mean values.

## Results

### Separation of ALDH^hi^CD44^+^ cells from MDA-MB-231 cell culture

**Figure 1:**
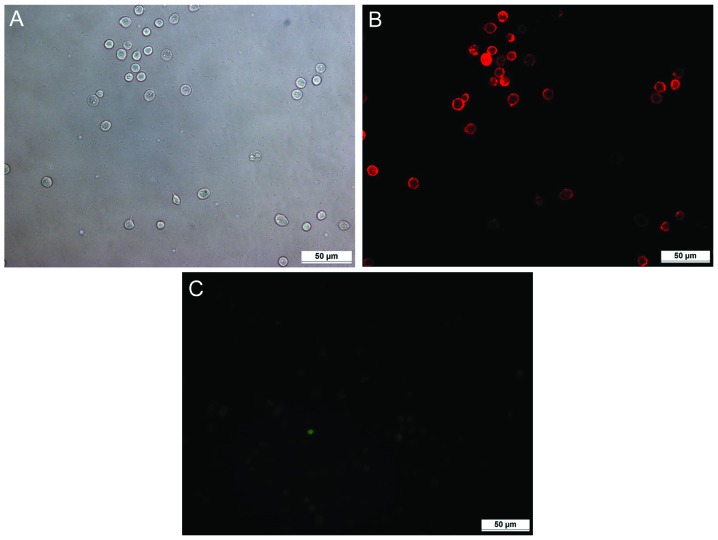
Proportion of cluster of differentiation (CD)44^+^ and acetaldehyde dehydrogenase (ALDH)^hi^ cells in an MDA-MB-231 cell suspension, detected using a fluorescence microscope (magnification, ×100). (A) MDA-MB-231 cell suspension. (B) High proportion of CD44^+^ cells (red fluorescence) and (C) low proportion of ALDH^hi^ cells (green fluorescence).

**Figure 2:**
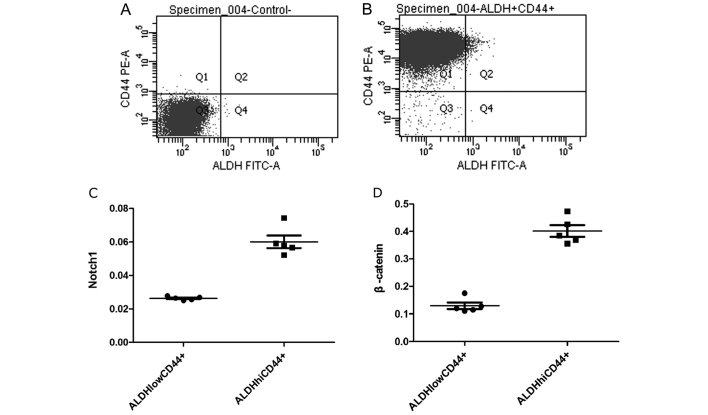
Results of flow separation experiment and expressions of activated Notch1 and β-catenin genes in stem-like ALDH^hi^CD44^+^ and non-stem-like ALDH^low^CD44^+^ cells. (A) ALDH+diethylaminobenzaldehyde+immunoglobulin G1-PE(Q3); (B) proportion of ALDH^hi^CD44^+^(Q2) and ALDH^low^CD44^+^ cells (Q1) in the MDA-MB-231 cells. (C) The Notch1 gene; and (D) the β-catenin gene. GAPDH was used as a reference gene. (C) and (D) represent the mean cycle threshold values of five independent experiments. ALDH, acetaldehyde dehydrogenase; CD, cluster of differentiation; PE, phycoerythrin; FITC, fluorescein isothiocyanate.

Using fluorescence microscopy, it was identified that the proportion of CD44^+^ cells in the MDA-MB-231 cell culture was high and that the proportion of ALDH^hi^ cells was comparably low (Fig. 1). Thus, consistent with a previous study (19), the content of stem-like ALDH^hi^CD44^+^ cells in the MDA-MB-231 cells was 0.82±0.067%, as determined by flow cytometry (Fig. 2A and B). In the present study, ~1×10^4^ ALDH^hi^CD44^+^ and ALDH^low^CD44^+^ cells were obtained from a culture of ~1×10^7^ MDA-MB-231 cells using a flow sorter for subsequent analysis by qPCR.

### Notch1 and β-catenin mRNA expression in stem-like ALDH^hi^CD44^+^ and non-stem-like ALDH^low^CD44^+^ cells

The mRNA expression levels of Notch1 and β-catenin were detected by performing qPCR (Fig. 2C and D). The mean Ct, ΔCt and 2^−ΔCt^ values were utilized to analyze gene expression in the two types of cell (Tables I and II), with a smaller Ct value indicating higher objective gene expression. Notch1 and β-catenin mRNA expression levels were identified in the ALDH^hi^CD44^+^ and ALDH^low^CD44^+^ cells, however, the mRNA expression level was significantly higher in the ALDH^hi^CD44^+^ cells compared with the ALDH^low^CD44^+^ cells (P<0.05), illustrating that the Notch and Wnt signaling pathways were highly expressed in the stem-like ALDH^hi^CD44^+^ cells.

### Proportion of ALDH^hi^CD44^+^ cells in breast cancer tissues

**Figure 3:**
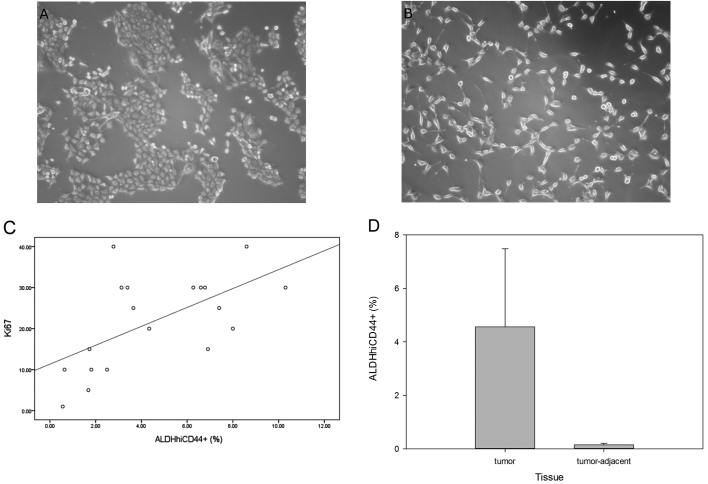
Contents of the primary culture of breast tumor and tumor-adjacent tissues analyzed using flow cytometry, and the correlation between the abundance of stem-like cells and the immunohistochemical staining results. Microscopy of the (A) breast tumor and (B) tumor-adjacent tissues (magnification, ×100). (C) Correlation between the ALDH^hi^CD44^+^ cells and Ki-67 expression levels. (D) The expression of the ALDH^hi^CD44^+^ cells was significantly higher in the tumor tissues compared with the tumor-adjacent tissues (P<0.05). ALDH, acetaldehyde dehydrogenase; CD, cluster of differentiation.

Successful primary culturing was achieved in 20/24 cases of invasive breast cancer tissues and 14/17 cases of healthy cancer-adjacent tissues (Fig. 3A and B). The average proportion of ALDH^hi^CD44^+^ cells was 4.56±2.92% (range, 0.56–10.30%) in the tumor tissues and 0.15±0.06% (range, 0.05–0.23%) in the tumor-adjacent tissues. Therefore, the expression level of the ALDH^hi^CD44^+^ cells in the cancer tissues was significantly higher than that in the adjacent tissue samples (P<0.05; Fig. 3D).

### Correlation between ALDH^hi^CD44^+^ expression and IHC staining

All 10% formalin-fixed and paraffin-embedded breast carcinoma tissue blocks were analyzed by two independent pathologists from Peking University Shenzhen Hospital. The principle IHC steps include deparaffinization in xylene and rehydration through a graded ethanol series, antigen retrieval, and incubation of serum proteins and primary antibodies in a fridge overnight. Finally, coloration of the secondary antibodies in DAB was performed for 10–15 min. Cancer cells without coloring demonstrated negative expression, and ER, PR and HER2 protein expression levels were assessed as positive if IHC staining was strong (+++), equivocal for IHC (++), and negative for IHC (− and +), in accordance with the American Society of Clinical Oncology/College of American Pathologists guidelines (20). Furthermore, FISH was used to perform genetic HER2 testing on tissues scored as borderline (++) by IHC analysis. The Ki-67 labeling index of the patients’ specimens were determined by two pathologists (Table III). No significant difference (P>0.05) was identified between the ALDH^hi^CD44^+^ cells, and ER (P=0.065), PR (P=0.107) and HER2 (P=0.050) expression levels; however, a significant difference (P<0.05) was identified between the ALDH^hi^CD44^+^ cells and Ki-67 expression (P=0.007) (Fig. 3C).

## Discussion

Radiotherapy, chemotherapy and endocrine therapy remain the primary treatment strategies for breast cancer patients prior to and following surgery, however, resistance to these treatments has recently become a major clinical issue (1–3). Considerable efforts have been made to elucidate the various distinct mechanisms that may cause relapse and therapy failure. Increasing evidence supports the hypothesis that the resistance arises from cancer stem-like cells, which can survive drug therapy and develop into new tumors. Recent studies have focused on the associated cell signaling pathways and the drug resistance proteins expressed in these stem-like cells. For example, Tanei *et al* (21) proposed that ALDH1, which appears to affect the recurrence and survival rates of breast cancer patients, may be associated with tumor resistance to chemotherapy. Furthermore, according to Croker and Allan (7), numerous drug resistance proteins demonstrate markedly higher expression levels in ALDH^hi^CD44^+^ cells compared with ALDH^low^CD44^−^ cells.

In the present study, ALDH^hi^CD44^+^ and ALDH^low^CD44^+^ cell subsets were obtained from MDA-MB-231 cells using flow separation technology, and qPCR was utilized to assess the mRNA expression levels of Notch1 and β-catenin. It was identified that these two important cell signaling genes were expressed in the stem-like ALDH^hi^CD44^+^ cells, and that β-catenin and Notch1 were expressed at a significantly higher level in the stem-like ALDH^hi^CD44^+^ cells compared with the non-stem-like ALDH^low^CD44^+^ cells (P<0.05). This indicated that the Wnt and Notch cell signaling pathways are activated in stem-like ALDH^hi^CD44^+^ cells. Rizzo *et al* (22) determined that interference RNA or specific secretion enzyme inhibitors could be used to markedly reduce Notch1 activation and effectively enhance the sensitivity of breast cancer cells to tamoxifen. Additionally, Zang *et al* (23) clarified that the downregulation of Notch1 activity increased the sensitivity of breast cancer cells to chemotherapy. One of the central aims of the stem cell biology of drug resistance is to understand the molecular mechanisms that control self-renewal in cancer stem cells. The results of the present and aforementioned studies indicate that the fate of stem cells may be determined by altering the signal transduction pathways associated with the self-renewal capacity of the cells.

The protein expression levels of ER, PR, HER2 and Ki-67 have a prognostic and predictive value for breast cancer patients (24). Ki-67 is a protein present in the nucleus of proliferating cells that is required to regulate cell proliferation, and the Ki-67 labeling index is chiefly important for distinguishing different breast cancer subtypes, such as luminal A and luminal B breast cancer cells (16). Furthermore, Ki-67 is an important predictive marker for determining the chemotherapeutic efficacy of breast cancer. However, the currently used method of cell counting for assessing Ki-67 expression levels lacks objectivity and is inconvenient for clinical pathologists without the availability of a standardized system. In the present study, it was detected that the presence of stem-like ALDH^hi^CD44^+^ cells was significantly associated with a high Ki-67 score. Previous studies have determined that breast cancer patients with high Ki-67 scores had poorer relapse-free survival rates than patients with low Ki-67 scores (25), therefore, breast cancer stem-like cells may potentially be associated with Ki-67 expression, providing a novel assessment method for pathologists to evaluate the Ki-67 scores, as opposed to counting positive and negative cells (26,27). However, the cohort of patients analyzed in the present study was limited. Thus, our future study will enroll a larger cohort of patients with breast cancer, grouped as previously described (low, ≤10% Ki-67-positive cells; intermediate, >10≤20% Ki-67-positive cells; and high, >20% Ki-67-positive cells) (28), to specifically evaluate the association between different Ki-67 protein expression levels and stem-like cells.

In conclusion, the present study identified that two cell signaling pathways that are important in drug resistance, Notch and Wnt/β-catenin, were abnormally activated in stem-like ALDH^hi^CD44^+^ cells compared with non-stem-like ALDH^low^CD44^+^ cells. Furthermore, a high correlation was identified between breast cancer stem cells and Ki-67 expression, however, no correlation was observed with ER, PR or HER2 expression; therefore, ALDH^hi^CD44^+^ cells may serve as novel diagnostic and prognostic factors in breast cancer.

## Figures and Tables

**Table I:** Mean Ct values of ALDH^hi^CD44^+^ and ALDH^low^CD44^+^ cells, as determined by quantitative polymerase chain reaction.

	Ct value, mean ± standard deviation			
	
	Notch1		β-catenin	
		
Time	ALDH^low^CD44^+^	ALDH^hi^CD44^+^	ALDH^low^CD44^+^	ALDH^hi^CD44^+^
I	29.71±0.04	27.31±0.19	27.51±0.18	24.79±0.24
II	29.62±0.65	27.50±0.15	26.81±0.49	24.32±0.09
III	29.74±0.18	27.54±0.07	27.58±0.36	24.89±0.01
IV	29.74±0.05	27.54±0.19	27.58±0.16	24.87±0.06
V	29.53±0.16	27.58±0.20	27.47±0.22	24.88±0.02

Ct, cycle threshold; ALDH, acetaldehyde dehydrogenase; CD, cluster of differentiation.

**Table II:** 2^−ΔCt^ values of ALDH^hi^CD44^+^ and ALDH^low^CD44^+^ cells, as determined by quantitative polymerase chain reaction.

	2^−ΔCt^ value			
	
	Notch1		β-catenin	
		
Time	ALDH^low^CD44^+^	ALDH^hi^CD44^+^	ALDH^low^CD44^+^	ALDH^hi^CD44^+^
I	0.027776	0.074325	0.127627	0.426317
II	0.025033	0.052193	0.175556	0.473029
III	0.026830	0.056720	0.119908	0.356013
IV	0.025559	0.057912	0.114229	0.368567
V	0.026461	0.059129	0.110338	0.384219

(ΔCt = Ct1 − Ct0, where Ct1 is the objective gene and Ct0 is GAPDH). CT, cycle threshold; ALDH, acetaldehyde dehydrogenase; CD, cluster of differentiation.

**Table III:** Contents of ALDH^hi^CD44^+^ cells in primary cultured cells from the tumor tissues, and expression of ER, PR, HER2 and Ki-67 in 19 patients with breast cancer.

		Protein expression levels			
		
Patient no.	ALDH^hi^CD44^+^ cells, %	ER	PR	HER2	Ki-67, %
1	2.50	+++	+	+	10
2	1.81	+++	+++	+	10
3	8.60	+	+	+++	40
4	6.91	++	+	−	15
5	8.00	−	−	+++	20
6	7.40	+++	++	+	25
7	3.13	+	+	+++	30
8	1.73	++	++	+	15
9	3.39	++	+	+	30
10	10.30	++	+	++	30
11	1.68	+	+	++	5
12	0.64	+++	+++	+	10
13	6.27	+	+	+++	30
14	2.78	+++	+	+++	40
15	6.60	+	+	+++	30
16	0.56	+++	+	+	1
17	3.65	+	+	+	25
18	4.34	++	++	+	20
19	6.78	+	+	+++	30

ALDH, acetaldehyde dehydrogenase; CD, cluster of differentiation; ER, estrogen receptor; PR, progesterone receptor; HER2, human epidermal growth factor receptor-2.

## Abbreviations

ALDH1: acetaldehyde dehydrogenase 1
ER: estrogen receptor
PR: progesterone receptor
HER2: human epidermal growth factor receptor 2
FBS: fetal bovine serum
IHC: immunohistochemical
FISH: fluorescence *in situ* hybridization
